# Adaptive capacity to dietary Vitamin B12 levels is maintained by a gene‐diet interaction that ensures optimal life span

**DOI:** 10.1111/acel.13518

**Published:** 2021-12-08

**Authors:** Tripti Nair, Rahul Chakraborty, Praveen Singh, Sabnam Sahin Rahman, Akash Kumar Bhaskar, Shantanu Sengupta, Arnab Mukhopadhyay

**Affiliations:** ^1^ Molecular Aging Laboratory National Institute of Immunology New Delhi India; ^2^ CSIR‐Institute of Genomics and Integrative Biology New Delhi India; ^3^ Academy of Scientific and Innovative Research (AcSIR) Ghaziabad India

**Keywords:** *Caenorhabditis elegans*, *flr‐4*, gene expression, life span, one‐carbon metabolism, osmotic stress, p38‐MAPK, Vitamin B12

## Abstract

Diet regulates complex life‐history traits such as longevity. For optimal lifespan, organisms employ intricate adaptive mechanisms whose molecular underpinnings are less known. We show that *Caenorhabditis elegans* FLR‐4 kinase prevents lifespan differentials on the bacterial diet having higher Vitamin B12 levels. The *flr*‐*4* mutants are more responsive to the higher B12 levels of *Escherichia coli* HT115 diet, and consequently, have enhanced flux through the one‐carbon cycle. Mechanistically, a higher level of B12 transcriptionally downregulates the phosphoethanolamine methyltransferase *pmt*‐*2* gene, which modulates phosphatidylcholine (PC) levels. *Pmt*‐*2* downregulation activates cytoprotective gene expression through the p38‐MAPK pathway, leading to increased lifespan only in the mutant. Evidently, preventing bacterial B12 uptake or inhibiting one‐carbon metabolism reverses all the above phenotypes. Conversely, supplementation of B12 to *E*. *coli* OP50 or genetically reducing PC levels in the OP50‐fed mutant extends lifespan. Together, we reveal how worms maintain adaptive capacity to diets having varying micronutrient content to ensure a normal lifespan.

## Graphical abstract

Multiple genes interact to maintain physiological homeostasis when *Caenorhabditis elegans* feeds on the varied diet it encounters in its ecological niche. Worms harboring a kinase‐dead version of the FLR‐4 protein are more responsive to the higher dietary Vitamin B12 present in *Escherichia coli* HT115. This boosts the flux through the one‐carbon metabolism, leading to reduction in phosphatidylcholine levels. Since the mutant FLR‐4 fails to prevent p38‐MAPK activation, these animals have better health and life span.

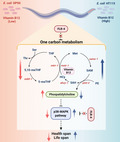

**Abbreviations**
[(S)PNPA]: (S)‐N‐palmitoyl‐norleucinol 1 phosphate
B12: Vitamin B12
CyTP: Cytoprotective
GFP: Green Fluorescent Protein
Hcy: Homocysteine
LPA: Lysophosphatidic acid
Met: Methionine
NHR: Nuclear Hormone Receptor
PC: Phosphatidylcholine
PE: Phosphatidylethanolamine
qRT‐PCR: Quantitative Real‐time PCR
RNAi: RNA interference
SAH: S‐adenosylhomocysteine
SAM: S‐adenosylmethionine

## 1 INTRODUCTION

The ability to forage on a wide range of diets is evolutionarily advantageous as organisms would flourish even when their preferred diet is depleted. Nutritional inputs play an indispensable role in maintaining cellular activities, and as a result in sustaining all life‐history traits such as development and reproduction. Nutritional availability also has a significant impact on the health and aging of an organism (Maier et al., 2010). Diet generally comprises of macronutrients but is also an important source for micronutrients such as vitamins that function as metabolic cofactors. As animals are exposed to food of various nutritional qualities, they have evolved intricate mechanisms to maintain homeostasis and normal life‐history traits, including life span, in response to varied dietary cues, although the underlying molecular mechanisms are less explored.

The free‐living bacterivorous nematode *Caenorhabditis elegans* has been widely used in understanding the role of nutrient signaling in aging (Kenyon, 2005, 2010; Kenyon et al., 1993). Extensive research in *C*. *elegans* has dissected the mechanisms of longevity assurance when nutrient availability is constrained by Dietary Restriction (DR) (Bishop & Guarente, 2007; Fontana et al., 2010; Greer & Brunet, 2009; Greer et al., 2007; Kaeberlein et al., 2006; Kapahi et al., 2017; Kenyon, 2010; Klapper et al., 2016; Lakowski & Hekimi, 1998; Mair & Dillin, 2008; Pang & Curran, 2014; Panowski et al., 2007; Toth et al., 2008; Walker et al., 2005; Wu et al., 2019). Recent research has also started to elucidate how micronutrients regulate life‐history traits of worms, including longevity (Bito et al., 2013; Bito & Watanabe, 2016; Maynard & Weinkove, 2020; Virk et al., 2012, 2016). This is primarily because the metabolically active intestinal microbiota of *C*. *elegans*, a major source of micronutrients, provides a relatively less complicated and genetically well‐regulated model to analyze the direct as well as bacterial feed‐mediated effects of diet on life span.

Although *C*. *elegans* feeds on a wide range of bacterial diets, they can maintain normal life‐history traits on most. This adaptive capacity to different diets is maintained by genes that have mostly been identified serendipitously (Maier et al., 2010; Mizunuma et al., 2014; Pang & Curran, 2014). When any of these genes are mutated, the worms fail to maintain homeostasis in various aspects of cellular physiology and start displaying altered life‐history traits on one diet and not the others. These “gene‐diet pairs” have been instrumental in our understanding of how the quality of food influences life span and health (Maier et al., 2010; Mizunuma et al., 2014; Pang & Curran, 2014).

Recently, we serendipitously discovered a diet‐gene pair where a serine–threonine‐specific kinase gene (*flr*‐*4*) mutant displayed a diet‐responsive increase in life and health span (Kobayashi et al., 2011; Take‐uchi et al., 2005; Verma et al., 2018). The kinase‐dead *flr*‐*4(n2259)* [*flr*‐*4(*−*)*] lives longer when grown on *Escherichia coli* HT115 but not on *E*. *coli* OP50. In *flr*‐*4(n2259)* grown on HT115, the p38‐MAPK pathway was found to be activated, leading to higher expression of cytoprotective (CyTP) xenobiotic detoxification genes through the Nuclear Hormone Receptor‐8 (NHR‐8) transcription factor. This suggested that *flr*‐*4(*−*)* mutants become responsive to the presence of a molecule(s) in HT115 to mount a specific response, whereas wild‐type worms can maintain homeostasis.

In this study, we identify Vitamin B12 or cobalamin as the micronutrient in *E*. *coli* HT115 that *flr*‐*4(*−*)* responds to, extending life span and increasing stress tolerance. We show that that *flr*‐*4(*−*)* worms are more responsive to the higher Vitamin B12 content of HT115. Consequently, these worms have increased flux through the one‐carbon metabolism and transcriptional downregulation of the phosphoethanolamine methyltransferase gene *pmt*‐*2* that is responsible for the biosynthesis of phosphatidylcholine (PC) from phosphatidylethanolamine (PE). We show that altering the levels of PC in *flr*‐*4(*−*)* worms, either genetically or by diet supplements, activates cytoprotective genes, increases stress tolerance and life span through the p38‐MAPK. Together, the serine–threonine kinase FLR‐4 maintains adaptive capacity towards a diet of different Vitamin B12 levels to preserve normal life‐history traits of *C*. *elegans*.

## 2 RESULTS

### 2.1 *Flr‐4(n2259)* is more responsive to Vitamin B12

**FIGURE 1:**
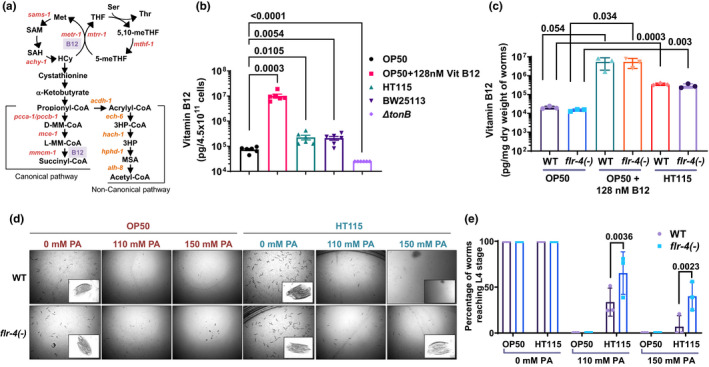
*Flr*‐*4(n2259)* is more responsive to higher Vitamin B12 content of *Escherichia coli* HT115. (a) A simplified view of the One‐carbon metabolism in *Caenorhabditis elegans*, comprising of the folate, methionine and propionate (canonical and non‐canonical) metabolic pathways. The metabolites are marked in black. The METR‐1 and MMCM‐1 enzymes use B12 as cofactors. (b) The B12 levels of OP50, HT115, OP50 supplemented with B12, BW25113 and *∆tonB*. Data presented as mean value of six biologically independent experiments ± SEM. *p*‐values determined by unpaired two‐tailed *t*‐test. (c) Diet‐dependent increase in B12 levels in wild‐type and *flr*‐*4(*−*)* fed OP50, OP50 supplemented with B12, and HT115. Data presented as mean value of three biologically independent experiments ± SD. *p*‐values determined by multiple *t*‐test using the Holm‐Sidak method. (d) Wild‐type and *flr*‐*4(*−*)* animals were subjected to propionate toxicity assay. L1 synchronized worms were grown on OP50 or HT115 supplemented with indicated concentrations of sodium propionate and checked for developmental arrest 72 h post‐L1. With increasing concentrations of propionate, both wild‐type and *flr*‐*4(*−*)* worms fed OP50 showed developmental arrest. Upon feeding HT115 with increasing concentrations of propionate, *flr*‐*4(*−*)* but not wild‐type reached adulthood, suggesting that the *flr*‐*4(*−*)* is more responsive to Vitamin B12. One of three biologically independent experiments shown. (e) Quantification of propionate toxicity assay in (d) using three independent experiments. Error bars are mean ± SD. *p*‐value determined using two‐way ANOVA with Sidak multiple comparisons test. Experiments were performed at 20°C. Source data is provided as a source data file. SD‐Standard Deviation; SEM‐ Standard Error Mean. PA‐ propionate

We sought to identify the key regulators originating from the bacterial diet that positively influence the life span of *flr*‐*4(*−*)* worms. We have observed that several genes functionally linked to the one‐carbon metabolism, for example, cysteine, taurine, methionine, glutamate as well as glutathione metabolism genes, are significantly upregulated in *flr*‐*4(*−*)* grown on HT115 (Verma et al., 2018). Importantly, two enzymes linked to the one‐carbon metabolism (METR‐1 and MMCM‐1) require Vitamin B12 (B12) as a cofactor (Figure 1a). Since worms depend on the bacteria for the supply of B12, we investigated if the differences in these micronutrient levels in HT115 and OP50 dictate the life span of the *flr*‐*4* mutant.

We directly quantified the levels of B12 in the OP50 and HT115 bacterial strains using a competitive intrinsic factor binding‐based assay and found that HT115 has significantly higher levels of B12 compared to OP50 (Figure 1b). Like mammals, *C*. *elegans* are unable to synthesize B12 and depends solely on the bacteria for its supply (Maynard & Weinkove, 2020). Given the higher levels of B12 in HT115, we next tested whether internal levels of B12 in worms also vary accordingly. As expected, we observed significantly higher B12 levels in worms grown on HT115, compared to OP50 (Figure 1c). The levels were increased several folds more when the bacterial diet was externally supplemented with B12 (Figure 1b,c).

Using an alternate cell biology strategy, we assessed B12 levels in the *flr*‐*4(*−*)* by monitoring the GFP expression of the *Pacdh*‐*1*::*gfp* reporter strain. *Acdh*‐*1*, along with *ech*‐*6*, *hach*‐*1*, *hphd*‐*1* and *alh*‐*8*, is a part of a non‐canonical B12‐independent pathway that metabolizes one‐carbon metabolism intermediate, propionic acid, a toxic intermediate, into acetyl CoA (Figure 1a). As such, this pathway is activated in the absence or by low levels of B12; otherwise, propionic acid is converted to succinyl‐CoA through the canonical B12‐dependent pathway. The expression of *Pacdh*‐*1*::*gfp* transcriptional reporter faithfully reports the levels of B12, with high expression corresponding to reduced, and low expression to high B12 levels in worms (Watson et al., 2014, 2016). We grew *Pacdh*‐*1*::*gfp* and *flr*‐*4(*−*)*;*Pacdh*‐*1*::*gfp* strains on OP50 or HT115 and visualized GFP fluorescence. We found that *acdh*‐*1* expression was significantly high on OP50 compared to HT115, confirming our biochemical quantification that HT115 has higher levels of B12 (Figure S1a). The GFP expression was significantly suppressed when worms were grown on OP50 supplemented with B12. We also validated this by quantifying mRNA levels of *acdh*‐*1* and *hphd*‐*1* (Figure S1b).

We found that HT115 has more B12 compared to OP50, but the B12 levels in the wild‐type and *flr*‐*4(*−*)* are comparable when grown on the B12‐rich diets (either HT115 or OP50 supplemented with 128 nM B12). So, the phenotypes of *flr*‐*4(*−*)* reported earlier, that is, increased CyTP expression and longer life span on HT115, may not simply be explained by the differences in the levels of B12 in the bacteria. The question would arise as to why wild‐type worms also do not benefit from the higher levels of B12. One way of explaining the observation is by surmising that *flr*‐*4(*−*)* may be more responsive to the levels of B12 in the bacteria. To address this, we compared the expression of *acdh*‐*1* and *hphd*‐*1* in wild‐type and *flr*‐*4(*−*)* grown on OP50 (Figure S1b). We found that *flr*‐*4(*−*)* have lower *acdh*‐*1* and *hphd*‐*1* levels on OP50, compared to wild‐type, suggesting that these worms are more responsive to B12.

Responsiveness to B12 may also be measured by analyzing the ability of the wild‐type and mutant worms to detoxify externally supplemented propionate when fed either OP50 or HT115. We grew the wild‐type or *flr*‐*4(*−*)* on increasing quantities of propionic acid, a toxic metabolite, on OP50 or HT115 diet and determined whether the worms are developmentally arrested, or their growth is slowed down. We found that both wild‐type and mutant worms grown on HT115 are more resistant to 110 mM propionic acid, compared to the worms grown on OP50, showing again that the latter has a lower concentration of B12 (Figure 1d,e). On further increasing the concentration of propionic acid to 150 mM, *flr*‐*4(*−*)* worms on HT115 still produced viable offspring that developed through the larval stages while those on OP50 were mostly arrested at L1 (Figure 1d,e). These experiments show that the mutant worms are more responsive to the presence of B12 in HT115 and can benefit from it in terms of mounting a better propionate detoxification response through the canonical pathway.

### 2.2 Vitamin B12 influences CyTP gene expression in *flr‐4(n2259)*

**FIGURE 2:**
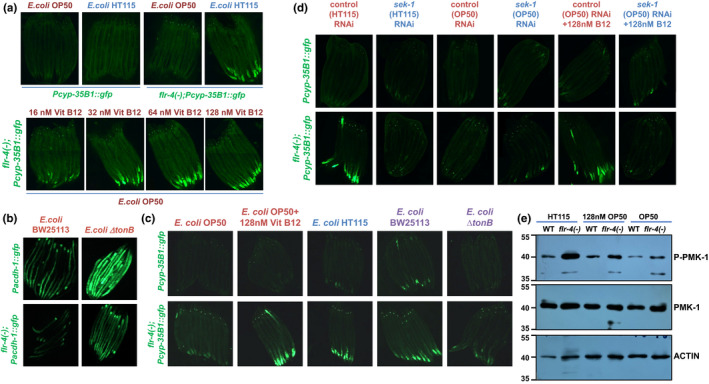
The expression of a cytoprotective (CyTP) gene in *flr*‐*4*(*n2259*) is regulated by dietary Vitamin B12. (a) The expression of *gfp* in *flr*‐*4(*−*)*;*Pcyp*‐*35B1*::*gfp* grown on OP50 was enhanced with increasing concentrations of B12. Quantification provided in Figure S2a,b. One of three biologically independent replicates shown. (b) The expression of *gfp* in *acdh*‐*1*::*gfp* and *flr*‐*4(*−*)*;*acdh*‐*1*::*gfp* increased when grown on *Escherichia coli ∆tonB* mutant as compared to the parent strain BW25113. The expression of *gfp* is lower in case of *flr*‐*4(*−*)*;*acdh*‐*1*::*gfp* compared to *acdh*‐*1*::*gfp* on either bacteria, suggesting that the *flr*‐*4(*−*)* is more responsive to B12. One of two biologically independent replicates shown. (c) The expression of *gfp* in *flr*‐*4(−)*;*Pcyp*‐*35B1*::*gfp* is higher compared to *Pcyp*‐*35B1*::*gfp* when grown on BW25113, comparable to HT115, or OP50 supplemented with B12, but is suppressed on Δ*tonB*. Quantification provided in Figure S2c. (d) The higher expression of *flr*‐*4(−)*;*Pcyp*‐*35B1*::*gfp* when grown on HT115 or OP50 supplemented with B12 was suppressed when the strains were grown on *sek*‐*1* RNAi. Quantification provided in Figure S2d. (e) Representative western blot of wild‐type and *flr*‐*4(−)* worms grown on OP50, HT115 or OP50 supplemented with B12, and probed with anti‐phospho‐PMK‐1 (P‐PMK‐1), anti‐PMK‐1 or anti‐Actin antibodies. One of two biologically independent experiments shown. Experiments were performed at 20°C. Source data is provided as a source data file

Having established that HT115 has higher levels of B12 and that *flr*‐*4(*−*)* is more responsive to it, we asked whether these attributes can explain various phenotypes where *flr*‐*4(*−*)* differs from wild‐type in a bacterial feed‐dependent manner. We have previously shown that *flr*‐*4(*−*)* worms have activated p38‐MAPK pathway leading to increased expression of downstream CyTP genes that supports increased life span, only when fed HT115 (Verma et al., 2018). So, we asked whether supplementing OP50 with B12 will generate the same transcriptomic response. As a representative CyTP gene, we had earlier used the *Pcyp35B1*::*gfp* (*CY573*) transgenic line (Verma et al., 2018). We grew *Pcyp35B1*::*gfp* or *flr*‐*4(*−*)*;*Pcyp35B1*::*gfp* worms on HT115, OP50 as well as OP50 supplemented with different concentrations of B12. We found that supplementing B12 to OP50 led to increased expression of GFP in the *flr*‐*4(*−*)*;*Pcyp35B1*::*gfp*, similar to the HT115 feed (Figures 2a and Figure S2a,b).

To confirm that the increased CyTP response is indeed mediated by the B12 absorbed by the bacteria, we performed an additional experiment with the *E*.* coli ∆tonB* bacterial strain defective in micronutrient absorption (Revtovich et al., 2019). Since the *∆tonB* mutant is in a different genetic background, we first tested the parent *E*. *coli* BW25113 strain for the *Pacdh*‐*1*::*gfp* response. We found that the expression of GFP was low, comparable to HT115 (Figure 2b, upper left panel) suggesting that the parent strain has higher levels of B12, which was also experimentally validated (Figure 1b). The *Pacdh*‐*1*::*gfp* levels were high when the worms were grown on *∆tonB* (Figure 2b, upper right panel). Since *flr*‐*4(*−*)* is more responsive to B12, the *flr*‐*4(*−*)*;*Pacdh*‐*1*::*gfp* had lower GFP fluorescence (Figure 2b, lower panels). Next, we grew *Pcyp35B1*::*gfp* and *flr*‐*4(*−*)*;*Pcyp35B1*::*gfp* worms on OP50, HT115, BW25113 and *∆tonB*. We found that the expression of GFP was lower in *∆tonB* compared to BW25113 and HT115, comparable to OP50 (Figures 2c and Figure S2c). This experiment shows that if *E*. *coli* is defective in the uptake of B12 from the media, it is not able to provide benefits to the *flr*‐*4* mutant. Overall, these results confirm that levels of B12 govern transcriptional upregulation of beneficial CyTP in *flr*‐*4(*−*)*.

The increased expression of CyTP genes in *flr*‐*4(*−*)* grown on HT115 is due to the activation of the p38‐MAPK pathway (Verma et al., 2018). So, we asked whether the increased expression of GFP in *flr*‐*4(*−*)*;*Pcyp35B1*::*gfp* was due to p38‐MAPK activation by B12. For this, we supplemented OP50, expressing either the control RNAi or the *sek*‐*1* (the p38‐MAPKK gene) RNAi, with B12 and grew *Pcyp35B1*::*gfp* or *flr*‐*4(*−*)*;*Pcyp35B1*::*gfp* on it. We found that this increase in GFP fluorescence was abrogated on *sek*‐*1* RNAi; there was no difference observed in the case of *Pcyp35B1*::*gfp* (Figures 2d and Figure S2d). We performed pPMK‐1 western analysis and found that B12 supplementation to OP50 led to increased phosphorylation of p38‐MAPK, similar to when the mutant worms were fed HT115 (Figure 2e). Together, these results show that the higher levels of B12 in HT115 and OP50 supplemented with B12 lead to the activation of p38‐MAPK and consequent upregulation of the CyTP gene in *flr*‐*4(*−*)*.

### 2.3 Vitamin B12 rescues osmotic tolerance and life span of *flr‐4(n2259)* fed OP50 diet

**FIGURE 3:**
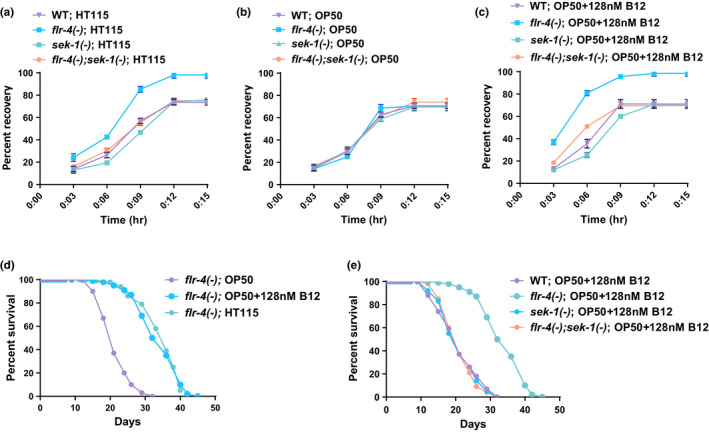
Vitamin B12 drives the increased osmotic tolerance and life span of *flr*‐*4(n2259)* grown on HT115, in a p38‐MAPK pathway‐dependent manner. (a) The *flr*‐*4(−)* worms are more osmo‐tolerant compared to wild‐type when grown on HT115. The *flr*‐*4(−)*;*sek*‐*1(km4)* worms are not tolerant to osmotic stress, showing that the increased osmotic tolerance of *flr*‐*4(−)* is dependent on the p38‐MAPK pathway. Osmotic stress was given to the worms at L4 stage by transferring them to 350 mM NaCl for 10 min. The recovery of the worms on NGM plates is plotted as a function of time. (b) No difference in osmotic tolerance is observed in the strains when grown on OP50. (c) The *flr*‐*4(−)* worms grown on OP50 supplemented with B12 is more osmotic tolerant compared to non‐supplemented worms. This increased tolerance is also dependent on the p38‐MAPK pathway as no osmotic tolerance was observed in *flr*‐*4(−)*;*sek*‐*1(km4)*. (d) The *flr*‐*4(−)* worms have increased life span only when grown on HT115 or B12‐supplemented OP50. (e) The *flr*‐*4(−)* worms have increased life span when grown on OP50 supplemented with B12. The supplementation was not able to increase life span in the *flr*‐*4(−)*;*sek*‐*1(km4)*. Life span and osmotic tolerance assays were performed at 20°C. One of three biologically independent experiment shown. Life span summary is provided in Table S1. Summary of osmotic tolerance assay is provided in Table S2. Experiments were performed at 20°C. Source data is provided as a source data file

Since B12 activates p38‐MAPK and enhances expression of the representative cytoprotective gene in *flr*‐*4(*−*)*, we asked if it improves the stress tolerance and life span of the mutant. We found that the mutant worms grown on HT115 are more resistant to osmotic stress and have better recovery kinetics, as compared to wild‐type (Figure 3a, Table S2 for details). The wild‐type and *flr*‐*4(*−*)* worms exhibit similar tolerance on OP50 (Figure 3b). The increased osmotic tolerance of *flr*‐*4(*−*)* was abrogated in *flr*‐*4(*−*)*;*sek*‐*1(km4)*, showing that it is p38‐MAPK‐dependent (Figure 3a). Finally, we asked whether supplementation of OP50 with B12 restores the ability of *flr*‐*4(*−*)* to resist osmotic stress. For this, we grew *flr*‐*4(*−*)* worms on OP50, HT115 and OP50 supplemented with B12. As expected, supplementation of B12 to OP50 restored the osmotic tolerance of the mutant to the levels obtained on feeding HT115, having little effect on the wild‐type (Figures 3c and Figure S3a,b). Also, *flr*‐*4(*−*)* grown on BW25113 were more osmotic tolerant compared to the B12‐deficient *∆tonB* mutant (Figure S3c). Importantly, the positive effect of B12 supplementation to OP50 on improved osmotic stress recovery of *flr*‐*4(*−*)* was also dependent on p38‐MAPK (Figure 3c).

Next, we supplemented the OP50 with B12 and conducted life span experiments, using worms grown on HT115 and OP50 without supplementation as controls. We found that the addition of B12 to OP50 completely rescued the life span of *flr*‐*4(*−*)* up to the levels of mutant worms grown on HT115 (Figure 3d, Table S1 for details). The wild‐type worms had no effect on life span on B12 supplementation (Figure S3d). Also, *flr*‐*4(*−*)* grown on BW25113 had an increased life span compared to the B12‐deficient *∆tonB* mutant (Figure S3e), confirming the involvement of B12. Importantly, the increased life span of *flr*‐*4(*−*)* on B12‐supplemented OP50 was dependent on the p38‐MAPK pathway (Figure 3e). Together, these experiments suggest that *flr*‐*4(*−*)* responds to the increased B12 levels in HT115 by activating the p38‐MAPK pathway to increase stress tolerance and prolong life span.

### 2.4 Enhanced flux through one‐carbon metabolism is essential for CyTP gene expression, stress tolerance and longevity of *flr‐4* mutant

**FIGURE 4:**
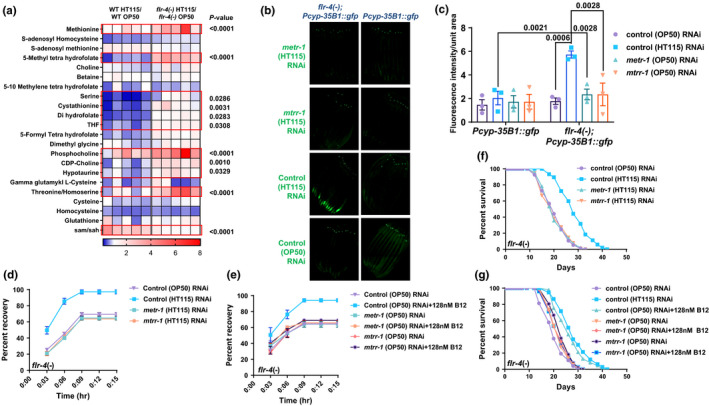
*Caenorhabditis elegans* one‐carbon metabolism catalyzes the effects of Vitamin B12 on *flr*‐*4(−)* gene expression, life span and osmotic tolerance. (a) Metabolomics analysis of wild‐type and *flr*‐*4(−)* grown on OP50 or HT115. Each box represents one of five biologically independent replicates. Differences are highlighted using red boxes. *p*‐value determined using two‐way ANOVA. (b) The increased expression of *gfp* that is observed in *flr*‐*4*(−);*Pcyp*‐*35B1*::*gfp* grown on control (HT115) RNAi (HT115 transformed with control RNAi vector), as compared to control (OP50) RNAi (OP50 transformed with control RNAi vector) is suppressed when the strain is grown on *metr*‐*1* or *mtrr*‐*1* (HT115) RNAi. One of three biological replicates. (c) Quantification of (b). Average of three biological replicates ± SEM. *p*‐value determined using two‐way ANOVA. (d) The increased osmotic tolerance of *flr*‐*4(−)* grown on control (HT115) RNAi is suppressed when the strain is grown on *metr*‐*1* or *mtrr*‐*1* (HT115) RNAi. One of three biologically independent experiments shown. (e) The increased osmotic tolerance of *flr*‐*4(−)* grown on control (OP50) RNAi supplemented with 128nM Vitamin B12 is suppressed when the strains are grown in *metr*‐*1* or *mtrr*‐*1* (OP50) RNAi background. One of three biologically independent experiments shown. (f) The increased life span of *flr*‐*4(−)* grown on control (HT115) RNAi is suppressed when *metr*‐*1* or *mtrr*‐*1* is knocked down using (HT115) RNAi. One of three biologically independent experiments shown. (g) The increased life span of *flr*‐*4(−)* grown on control (OP50) RNAi supplemented with 128 nM Vitamin B12 is suppressed when *metr*‐*1* or *mtrr*‐*1* is knocked down using (OP50) RNAi. One of three biologically independent experiments shown. All experiments were performed at 20°C. Life span summary is provided in Table S1. Summary of osmotic tolerance assay is provided in Table S2. Source data is provided as a source data file

We have shown that the higher B12 levels in HT115 combined with increased responsiveness to the micronutrient in *flr*‐*4(*−*)* leads to activation of the p38‐MAPK and increased CyTP gene expression. Since B12 is a major cofactor of the one‐carbon metabolism that is an important source of diverse metabolites, *flr*‐*4(*−*)* worms could benefit from the higher flux through this pathway when grown on HT115. We performed a metabolomics analysis and found that *flr*‐*4(*−*)* responded to B12 by enhancing the flux through the pathway, while wild‐type did not (Figure 4a). Interestingly, increased flux through One‐carbon metabolism in HT115‐fed *flr*‐*4(*−*)* seems to induce CyTP expression as we found that knocking down *metr*‐*1* or *mtrr*‐*1* (in HT115 background) abrogated the expression of GFP in *flr*‐*4(*−*)*;*Pcyp35B1*::*gfp*, without having any effect in *Pcyp35B1*::*gfp* (Figure 4b,c). Similarly, the increased expression of *gfp* in *flr*‐*4(*−*)*;*Pcyp35B1*::*gfp* grown on OP50 supplemented with 128 nM B12 was also suppressed on *metr*‐*1* and *mtrr*‐*1* (OP50) RNAi (Figure S4a). Importantly, the genes of one‐carbon metabolism were also required for osmotic tolerance (Figures 4d,e and Figure S4b,c) and life span enhancement (Figures 4f,g and Figure S4d,e) of *flr*‐*4(*−*)* grown on HT115 or OP50 supplemented with 128 nM B12. This shows that B12 from the bacteria may influence the one‐carbon metabolism in the *flr*‐*4* mutant, leading to activation of the p38‐MAPK pathway, impacting gene expression, osmotic tolerance and life span.

### 2.5 Vitamin B12 supplementation may activate p38‐MAPK in the *flr‐4* mutant by downregulating Phosphatidylcholine levels

**FIGURE 5:**
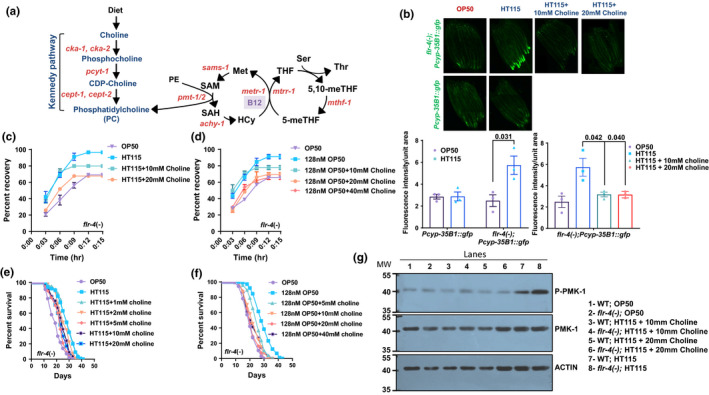
Benefits of high Vitamin B12 diet in *flr*‐*4* mutant is abrogated on choline supplementation. (a) An abridged view of the one‐carbon metabolism and Kennedy pathway in *Caenorhabditis elegans*. The genes coding for the enzymes in the pathway are marked in orange. The enzymes CKA‐1, CKA‐2, PCYT‐1, CEPT‐1 and CEPT‐2 convert dietary choline to phosphatidylcholine (PC) via Kennedy pathway. PC is also synthesized by PMT‐1 and PMT‐2 from phosphatidylethanolamine (PE) where S‐adenosylmethionine (SAM) acts as the methyl donor. 5,10‐meTHF, 5,10‐methyleneTHF; 5‐meTHF, 5‐methyl THF; HCy, homocysteine; Met, methionine; SAH, S‐adenosylhomocysteine; Ser, serine; THF, tetrahydrofolate; Thr, threonine. (b) The increased expression of *gfp* in *flr*‐*4*(−);*Pcyp*‐*35B1*::*gfp* grown on HT115 (as compared to OP50) is suppressed when the strain is grown on HT115 supplemented choline (10 or 20 mM). Quantification using three biologically independent experiment is shown below. Error bars are mean ± SEM. *p*‐value determined using unpaired *t*‐test. (c) The *flr*‐*4(−)* worms have increased osmotic stress tolerance when grown on HT115, as compared to OP50. The supplementation of choline (10 or 20 mM) to HT115 decreases osmotic stress tolerance of *flr*‐*4(−)*. (d) The *flr*‐*4(−)* worms have increased osmotic stress tolerance when grown on OP50 supplemented with B12, as compared to OP50. The supplementation of choline (10, 20 or 40 mM) decreases osmotic stress tolerance of *flr*‐*4(−)*. (e) The *flr*‐*4(−)* worms have increased life span when grown on HT115, as compared to OP50. When grown on HT115 supplemented with choline (1, 2, 5, 10 or 20 mM), the increased life span was suppressed. (f) The *flr*‐*4(−)* worms have increased life span when grown on OP50 supplemented with B12, as compared to OP50. When supplemented with choline (5, 10, 20 or 40 mM), the increased life span was suppressed. (g) Representative western blot of wild‐type and *flr*‐*4(−)* worms grown on OP50, HT115 or HT115 supplemented with choline and probed with anti‐phospho‐PMK‐1 (P‐PMK‐1), anti‐PMK‐1 or anti‐Actin antibodies. One of three biologically independent replicates shown for all experiments. Osmotic stress and life span assays were performed at 20°C. Life span summary is provided in Table S1. Summary of osmotic tolerance assay is provided in Table S2. Source data is provided as a source data file

Since B12 activates the flux through one‐carbon metabolism as well as the p38‐MAPK pathway in the *flr*‐*4* mutant, we investigated how these two pathways may be connected. It has been reported earlier that the depletion of Phosphatidylcholine (PC) levels activates p38‐MAPK pathway, although the mechanistic details are less known (Ding et al., 2015). PC biosynthesis occurs via two routes; (1) from dietary choline through the Kennedy pathway and (2) via tri‐methylation of PE by phosphoethanolamine methyltransferase PEMT/PMT‐1/2 (Figure 5a). We argued that if PC depletion is activating the p38MAPK‐pathway, and enhancing the expression of the downstream CyTP gene, osmotic stress tolerance and longevity, then supplementation of its upstream precursor choline should attenuate all these phenotypes. We monitored the GFP fluorescence of the CyTP reporter strain in *flr*‐*4(*−*)*;*cyp35B1*::*gfp* fed HT115 or B12 supplemented OP50 in the presence of choline. We found that choline supplementation reduced GFP fluorescence in both the conditions, almost to the levels of OP50 fed *flr*‐*4(*−*)*;*cyp35B1*::*gfp* (Figures 5b and Figure S5a). Additionally, choline supplementation suppressed increased osmotic stress tolerance and lifespan of *flr*‐*4* mutant fed HT115 or B12 supplemented OP50, without having any effect on wild‐type (Figure 5c–f, and Figure S5b–e). Further, using pPMK‐1 western, we also show that supplementation of choline suppressed the activation of the p38‐MAPK (Figure 5g).

**FIGURE 6:**
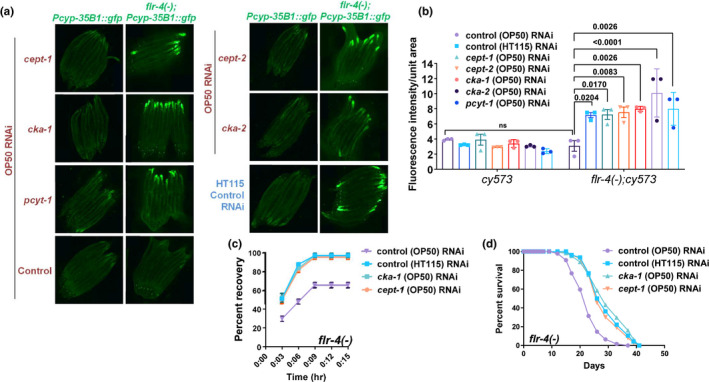
Reducing flux through Kennedy pathway activates cytoprotective genes and increases life span of *flr*‐*4(n2259)* even on *Escherichia coli* OP50. (a) The expression of *gfp* in *flr*‐*4(−)*;*Pcyp*‐*35B1*::*gfp* is increased when grown on control (HT115) RNAi, compared to control (OP50) RNAi. However, when these worms are grown on *cka*‐*1*, *cka*‐*2*, *pcyt*‐*1*, *cept*‐*1* and *cept*‐*2* in the OP50 RNAi system, *gfp* expression was elevated. (b) Quantification of (a). Average of three biological replicates ± SEM. *p*‐value determined using two‐way ANOVA with Tukey's correction. ns, non‐significant. (c) The osmotic stress tolerance of *flr*‐*4(−)* grown on control (HT115) RNAi is higher compared to control (OP50) RNAi. However, knocking down *cka*‐*1* and *cept*‐*1* using (OP50) RNAi leads to better osmotic stress tolerance of the mutant even in the OP50 feed. (d) The life span of *flr*‐*4(−)* grown on control (HT115) RNAi is higher compared to control (OP50) RNAi. Knocking down *cka*‐*1* and *cept*‐*1* using an OP50 RNAi system leads to longer life span even on the OP50 feed. One of three biologically independent replicates shown for all experiments. All experiments were performed at 20°C. Life span summary is provided in Table S1. Summary of osmotic tolerance assay is provided in Table S2. Source data is provided as a source data file

Alternatively, we validated our findings by testing if inhibiting choline synthesis activates the CyTP reporter in low B12 diet OP50. We used OP50 RNAi targeting *cept*‐*1*, *cept*‐*2*, *cka*‐*1*, *cka*‐*2* and *pcyt*‐*1* that would prevent the conversion of choline to PC. The RNAi inhibition resulted in upregulation of GFP in *flr*‐*4(*−*)*;*Pcyp35B1*::*gfp* almost to the level of HT115 fed worms; however, no change was observed in *Pcyp35B1*::*gfp* (Figure 6a,b). Under any of these conditions, choline supplementation did not suppress *flr*‐*4(*−*)*;*Pcyp35B1*::*gfp* expression as it is not likely to be converted to PC (Figure S6a). Importantly, knocking down of *cept*‐*1* or *cka*‐*1* in an *flr*‐*4(*−*)* background, using the OP50 RNAi system, significantly increased osmotic tolerance and life span; no effect was seen in the wild‐type (Figure 6c,d; Figure S6b,c). Together, these experiments show that high dietary B12 may lead to lower PC levels which in turn affects the cytoprotective gene expression, osmotic tolerance and life span of the *flr*‐*4* mutant.

### 2.6 Transcriptional downregulation of phosphoethanolamine methyltransferase gene *pmt‐2* on high Vitamin B12 diet

**FIGURE 7:**
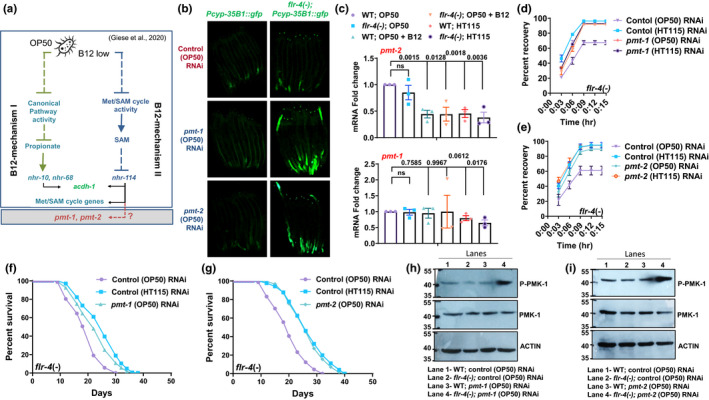
Expression of Phosphoethanolamine methyltransferase *pmt*‐*2* is transcriptionally downregulated in *flr*‐*4(n2259)* on high Vitamin B12 diet. (a) An abridged representation of the regulation of one‐carbon cycle highlighting the B12 mechanisms I and II, as described by the Walhout lab (Giese et al., 2020). Lower vitamin B12 levels in OP50 would lead to increased expression of methionine/SAM cycle genes. We hypothesized that this will also increase the expression of *pmt*‐*1* and/or *pmt*‐*2* (grey box). (b) Representative experiment to show that the expression of *flr*‐*4*(−);*Pcyp*‐*35B1*::*gfp* is increased on *pmt*‐*1 or pmt*‐*2* (OP50) RNAi. Quantification provided in Figure S7a,b. (c) RT‐PCR showing the expression levels of *pmt*‐*1* and *pmt*‐*2*. The expression of *pmt*‐*2* is sensitive to vitamin B12 levels. Average of three biological replicates ± SEM. Unpaired two‐tailed *t*‐test. ns, non‐significant. (d,e) The osmotic stress tolerance of *flr*‐*4(−)* grown on control (HT115) RNAi is higher compared to control (OP50) RNAi. However, knocking down (d) *pmt*‐*1* or (e) *pmt*‐*2* using (OP50) RNAi leads to better osmotic stress tolerance of the mutant even on the OP50 feed. (f,g) The life span of *flr*‐*4(−)* grown on control (HT115) RNAi is higher compared to control (OP50) RNAi. However, knocking down (d) *pmt*‐*1* or (e) *pmt*‐*2* using (OP50) RNAi leads to increased life span of the mutant even on the OP50 feed. (h) Representative western blot of wild‐type and *flr*‐*4(−)* worms grown on control (OP50) RNAi or *pmt*‐*1* (OP50) RNAi and probed with anti‐phospho‐PMK‐1 (P‐PMK‐1), anti‐PMK‐1 or anti‐Actin antibodies. (i) Representative western blot of wild‐type and *flr*‐*4(−)* worms grown on control (OP50) RNAi or *pmt*‐*2* (OP50) RNAi and probed with anti‐phospho‐PMK‐1 (P‐PMK‐1), anti‐PMK‐1 or anti‐Actin antibodies. One of three biologically independent replicates shown for western blots, *gfp*, osmo‐tolerance and life span experiments. All experiments were performed at 20°C. Life span summary is provided in Table S1. Summary of osmotic tolerance assay is provided in Table S2. Source data is provided as a source data file

Finally, we asked how higher B12 levels in HT115 may potentially lower PC levels. Recently, the Walhout lab has elucidated a novel gene regulatory mechanism (the B12 mechanism II) driven by B12 (Giese et al., 2020; Figure 7a). In response to low B12 levels (as in OP50 fed worms), the flux through the one‐carbon cycle is suppressed which lowers SAM levels. Low SAM levels activate the NHR‐114 transcription factor, which then transcribes one‐carbon cycle genes, as well as *acdh*‐*1*. We hypothesized that a low flux through the one‐carbon cycle may activate *pmt*‐*1*/*pmt*‐*2* and increase PC levels, which would suppress p38‐MAPK and CyTP expression. We grew *flr*‐*4(*−*)*;*Pcyp35B1*::*gfp* and *Pcyp35B1*::*gfp* on *pmt*‐*1* or *pmt*‐*2* (OP50) RNAi and found that GFP expression was increased (Figure 7b, quantification in Figure S7a,b). We also quantified transcript levels of *pmt*‐*1* and *pmt*‐*2* by qRT‐PCR. Importantly, we found that only the levels of *pmt*‐*2* were decreased on a higher B12 diet, while levels of *pmt*‐*1* remained unchanged (Figure 7c). Although B12 seems to affect mRNA levels of only *pmt*‐*2*, perturbing any of the two methyltransferases (*pmt*‐*1* or *pmt*‐*2*) may lower PC to activate CyTP expression. In line with these findings, knocking down *pmt*‐*1* and *pmt*‐*2* (using OP50 RNAi) increased osmotic stress tolerance as well as the life span of the *flr*‐*4(*−*)* (Figure 7d–g, Figure S7c–f). Further, knocking down *pmt*‐*2* or *pmt*‐*1* led to increased activation of p38‐MAPK (Figure 7h–i). Together, our data suggest that the B12 mechanism II may transcriptionally modulate PC levels in the *flr*‐*4* mutants grown on a B12‐rich diet, thereby increasing downstream CyTP gene expression, stress tolerance and life span, through activation of the p38‐MAPK pathway.

## 3 DISCUSSION

**FIGURE 8:**
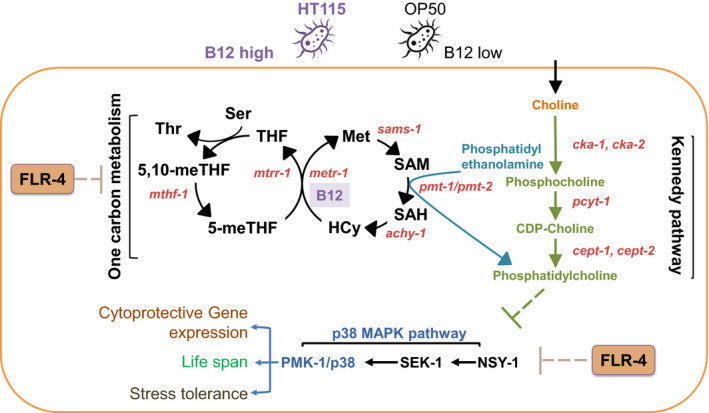
A model depicting the possible mechanism by which *C*. *elegans* FLR‐4 maintains adaptive capacity to bacterial diet differing in B12 content. On high B12 diet, flux through the one‐carbon metabolism increases to potentially lower PC levels; this leads to activation of the p38‐MAPK pathway. However, FLR‐4 prevents aberrant activation of the p38‐MAPK pathway leading to normal life span in wild‐type worms. In the *flr*‐*4(−)*, the lower PC levels activate the p38‐MAPK, increase CyTP gene expression, enhance osmotic tolerance and increase life span

In this study, we have shown that the worms maintain the adaptive capacity to diets of differing micronutrient content, specifically B12, by preventing ectopic activation of the p38 pathway. Our data suggest that in the *flr*‐*4* mutant worms, improved responsiveness to B12 coupled to increased availability of the micronutrient on feeding HT115 leads to elevated flux through the one‐carbon metabolism. Also, higher B12 results in a potential lowering of PC levels due to transcriptional downregulation of PMT‐2. Since FLR‐4 is active in wild‐type worms, ectopic activation of the p38‐MAPK is prevented, and life span homeostasis is maintained. However, the kinase‐dead *flr*‐*4* mutant is incapable of suppressing the p38‐MAPK pathway, resulting in increased downstream cytoprotective gene expression, enhanced osmotic tolerance and longer life span (Figure 8).

While we have placed FLR‐4 downstream of the one‐carbon cycle, negatively regulating the activity of the p38‐MAPK, it may genetically also act upstream or at the level of the cycle. This is supported by the observations that (1) the mutant shows lower *acdh*‐*1* expression compared to WT when grown on OP50, (2) mutants are more resistant to propionate, and (3) the one‐carbon metabolism flux is increased only in the mutant but not in wild‐type. Moreover, this responsiveness is not due to the higher uptake of B12 in the mutant as the micronutrient content is the same as in wild‐type. Since FLR‐4 is a kinase, the kinase‐dead mutant may fail to inhibit a critical enzyme of the pathway itself, thereby enhancing flux through the one‐carbon cycle. It is also possible that another small molecule of the bacteria in addition to B12 modulates the one‐carbon metabolism in the mutant. Future investigations need to be directed to answer these questions.

In *C*. *elegans*, like other organisms, B12 inputs from diet and microbial colonization of B12‐producing bacteria in the intestine regulate the flux through the one‐carbon metabolism, comprising of the methionine and folate metabolism arms, as well as modulate the clearance of propionate (Bito & Watanabe, 2016; Watson et al., 2014; Yilmaz & Walhout, 2014; Zhang et al., 2020). Due to the central nature of the one‐carbon metabolism, B12 is known to play a role in immune modulation, stress tolerance, fertility, development and memory (Bito et al., 2013, 2017; Candito et al., 2003; Pront et al., 2009; Revtovich et al., 2019; Schaffner et al., 2019; Verma et al., 2018; Watanabe et al., 2003; Yilmaz & Walhout, 2014; Yoshii et al., 2019; Zhang et al., 2020). While B12 deficiency affects pathogen response, lowers fertility, delays growth, and shortens lifespan (Bito & Watanabe, 2016; Ding et al., 2015), excess of the micronutrient accelerates growth, increases fecundity and shortens life span (Macneil & Walhout, 2013; Yilmaz & Walhout, 2014). Thus, the worms need to maintain normal life‐history traits on different diets with varying concentrations of B12. We show that FLR‐4 maintains an adaptive capacity to bacterial diet with varying concentrations of a micronutrient through an intricate gene‐diet pairing that influences the one‐carbon metabolism.

While the adaptive capacity to diet is an important homeostatic mechanism, only a handful of gene‐diet interactions have been characterized in detail. More importantly, the connections between these diverse genes that maintain homeostasis are scant. A *C*. *elegans* mutant of the mammalian neuromedin U receptor homolog, *nmur*‐*1* was previously shown to regulate life span dependent on food type; the mutant lives long on OP50, not on HT115 (Maier et al., 2010). NMUR‐1 works in the sensory neurons and helps the worms differentiate between the two bacteria depending on the lipopolysaccharide structure. A mutant of the proline metabolism pathway, on the other hand, accelerates aging on OP50 by producing mitochondrial damage due to the build‐up of reactive oxygen species and 1‐pyrroline‐5‐carboxylate (Pang & Curran, 2014; Yen & Curran, 2016). Intriguingly, the NMUR‐1 receptor pathway was required by the *alh*‐*6* mutant to shorten the life span on OP50. We have identified FLR‐4 as another gene that maintains adaptive capacity towards different diets (Verma et al., 2018). Interestingly, both ALH‐6 and FLR‐4 use mechanisms of longevity assurance that overlaps with that of dietary restriction. Recently, a mutation in the mitochondrial ribosomal gene *mrpl*‐*2* that mediates protein translation in mitochondria was found to activate the Unfolded Protein Response of the mitochondria (UPRmt) and promote longevity only when fed OP50. They also attributed this effect to the low availability of B12. It will be interesting to know whether this gene genetically interacts with FLR‐4. Further, the *rict*‐*1* mutants show differential life span, metabolism and development on different diets. The *rict*‐*1(*−*)* animals that were fed HT115 lived longer than wild‐type, but on OP50, their lifespan was significantly reduced (Mizunuma et al., 2014). We have found that when grown on *rict*‐*1* RNAi, the *flr*‐*4(*−*)* worms perish, suggesting a genetic interaction that needs to be characterized. Thus, a concerted effort needs to be made to identify more gene‐diet interactions as well as study the mechanistic crosstalks between the published pairs.

Several previous studies document inverse correlations between phosphatidylcholine levels and p38 activation. In *C*. *elegans*, activation of p38‐MAPK on *sams*‐*1* knockdown is suppressed if phosphatidylcholine levels are upregulated by the addition of choline (Ding et al., 2015). In IMR‐90 fibroblasts, Lysophosphatidic acid (LPA) analogues (S)‐N‐palmitoyl‐norleucinol 1‐phosphate [(S)PNPA] was found to induce apoptosis by specifically inhibiting PC biosynthesis, leading to p38 activation (Gueguen et al., 2002). Exogenous application of PC has been shown to have anti‐inflammatory properties in human Caco‐2 cells treated with tumour necrosis factor‐alpha (TNF‐alpha), by inhibiting p38 signalling (Treede et al., 2007, 2009). Our work also implicates the PC levels to lowering p38 activation. This raises a pertinent question as to how the PC: PE ratio may regulate the p38‐MAPK pathway. The reasons for this negative correlation need to be deciphered in future.

Diet‐gene interactions may provide an adaptive advantage to invertebrates like *C*. *elegans*, but such interactions may play important role in the pathophysiology of human diseases. For example, diet‐gene interplay may be the basis of the “copper phenotype” of sporadic Alzheimer's disease (AD) where a low copper diet reduces the risk of AD in individuals with altered copper metabolism (Squitti et al., 2014). Diet may also alter the impact of genetic variants on disease risk, as shown in the case of colorectal cancer where consumption of processed meat increased risk in individuals with rs4143094‐TG and ‐TT genotypes (Figueiredo et al., 2014). Genotypes of individuals studied in some randomized clinical trials have also been found to modify the effect of dietary interventions on weight loss, weight maintenance, lipid profile, insulin resistance, and blood pressure (Qi, 2014). This underscores the importance of directed studies to unravel more diet‐gene interactions and their underlying mechanisms, that are easier to study in model organisms.

## 4 EXPERIMENTAL PROCEDURE

Detailed experimental procedure is provided in Appendix S1.

*Caenorhabditis elegans* strains were maintained at 20°C on Nematode Growth Media (NGM) agar plates on *E. coli* OP50 bacterial lawns. All experiments were conducted with L1 synchronized worms. For RNAi plates, secondary cultures were concentrated 10 times in M9 buffer containing Ampicillin (100 mg/ml) and IPTG (1mM) before seeding plates supplemented with same amount of Ampicillin and IPTG. A B12 (V6629; Sigma) stock concentration was prepared using M9 buffer. The secondary bacterial cultures were concentrated 10 times in 1 × M9 buffer and desired concentration of B12 were added before seeding. Sodium propionate (P1880; Sigma) stock concentration of 1 M (pH 7) or Choline chloride (A15828; Alfa Aesar) stock concentration of 2 M was prepared in MilliQ water, filtered, and added to NGM agar. Life span analysis, RNA isolation, QRT‐PCR analysis, CyTP gene activation assay, western blotting analysis was performed as previously published (Chamoli et al., 2014; Verma et al., 2018). For Osmotic stress assay, L4 stage worms were exposed to 350 mM sodium chloride for 9 min and recovery was scored. For Propionate toxicity assay, L1 synchronized worms were placed on propionate‐supplemented plates and development was observed after 48 and 72 h. Intracellular B12 was measured by a kit‐based electrochemiluminescence immunoassay (ECLIA) using COBAS e411 Analyzer. Intracellular metabolites for MS‐based targeted metabolomics were extracted using cold Actonitrile–methanol–water and data were acquired using a Sciex Exion LCTM analytical UHPLC system coupled with a triple quadrupole hybrid ion trap mass spectrometer (QTrap 6500; Sciex) in a positive mode. Relative quantification was performed using MultiQuantTM software v.2.1 (Sciex). Cysteine and Homocysteine were measured by HPLC (Agilent 1290 Infinity II LC system).

## CONFLICT OF INTEREST

The authors declare no conflict of interest.

## AUTHOR CONTRIBUTIONS

AM and SS conceived the project. TN performed all experiments related to *Caenorhabditis elegans* and analyzed the data, along with SSR. RC, PS, AKB performed Vitamin B12 measurements and metabolomics experiments. AM and SS supervised the project. AM wrote the manuscript.

## Supporting information

Table S1

Table S2

Supplementary Material

## Data Availability

The data that supports the findings of this study are available in the supplementary material of this article. All data used in the manuscript is presented as a source data file.
