# Supplementary material for: Adaptive capacity to dietary Vitamin B12 levels is maintained by a gene‐diet interaction that ensures optimal life span
Source: Aging Cell. 2021 Dec 8;21(1):e13518. doi: 10.1111/acel.13518 (PMC8761004; doi:10.1111/acel.13518)
Supplement: Supplementary file 3 — Supplementary Material [file ACEL-21-e13518-s001.pdf]

Supplementary Information for

Adaptive capacity to dietary Vitamin B12 levels is maintained by a gene-diet interaction that ensures optimal life span

Tripti Nair[1], Rahul Chakraborty[2,3], Praveen Singh [2,3], Sabnam Sahin Rahman[1], Akash Kumar Bhaskar[2,3], Shantanu Sengupta[2] and Arnab Mukhopadhyay[1]*

[1]Molecular Aging Laboratory, National Institute of Immunology, Aruna Asaf Ali Marg, New Delhi 110067, India

[2] CSIR-Institute of Genomics and Integrative Biology, Mathura Road Campus, New Delhi 110025, India

[3]Academy of Scientific and Innovative Research (AcSIR), Ghaziabad 201002, India

*Corresponding author (arnab@nii.ac.in)

Detailed experimental procedure

C. elegans strains and maintenance

Caenorhabditis elegans strains were obtained from the Caenorhabditis Genetics Center and maintained at 20 °C on Nematode Growth Media (NGM) agar plates on *Escherichia coli* OP50 bacterial lawns. All experiments were conducted with L1 synchronized worms. Strains used are: N2 Bristol as wild-type, *flr-4*(*n2259*), *sek-1*(*km4*)X, *flr-4*(*n2259*)X; *sek-1*(*km4*)X, *Pcyp-35B1::gfp* i.e. *bvIs5* [*Pcyp-35B1::GFP* + *gcy-7p::GFP*], *flr-4*(*n2259*)X;*bvIs5*, *acdh-1p::GFP* i.e. *wwIs24* [*acdh-1p::GFP* + *unc-119(+)*] and *flr-4*(*n2259*)X;*wwIs24*.

Bacterial growth

Bacteria from glycerol stocks were streaked on Luria-Bertani plates and incubated overnight (16 h) at 37 °C till single colonies were distinctly visible. Single colony was inoculated in Luria-Bertani broth and grown overnight (12 h) at 37 °C for primary culture. The secondary cultures were inoculated with 1/100th volume of primary culture and incubated at 37 °C till the optical density (OD_{600}) reached 0.6. The secondary cultures were concentrated 10 times and 250 μL and 1ml of secondary cultures were plated on 60mm and 90 mm NGM plates, respectively. After seeding, the plates were dried, and the bacterial lawn were grown overnight before worms were placed on them. Following bacterial strains were used *E. coli* OP50, *E. coli* HT115, *E. coli* BW25113 and *E. coli* ΔtonB.

Feeding RNAi

Bacterial clones from glycerol stocks were streaked on Luria- Bertani plates and incubated overnight (16 h) at 37 °C. Single colony was inoculated in Luria-Bertani broth containing Ampicillin (100 mg/ml) and tetracycline (12.5 mg/ml). Primary cultures were grown overnight (12 h) at 37 °C. The secondary cultures were inoculated with 1/100th volume of primary culture and incubated at 37 °C till the optical density (OD_{600}) reached 0.6. The secondary cultures were concentrated 10 times in M9 buffer containing Ampicillin (100 mg/ml) and IPTG (1mM). Secondary cultures of volume 250 μl and 1ml

were plated on 60mm and 90 mm NGM plates, respectively, that were also supplemented with Ampicillin (100 mg/ml) and IPTG (1mM).

Synchronization of animals

Gravid worms, grown on *E. coli* OP50, were bleached and eggs were subjected to L1 starvation for 16 hours in M9 buffer at 20 ^{o}C. L1 larvae were centrifuged at 2500 rpm for one minute, the buffer was aspirated and the L1 larvae were placed on desired experimental or maintenance plates.

Vitamin B12 supplementation

A vitamin B12 (V6629; Sigma, USA) stock concentration of 3 mM was prepared using M9 buffer. Further, a working stock of 1 mM was prepared by diluting the 3 mM stock with 1 x M9 buffer and filtered. The secondary cultures were concentrated 10 times in 1 x M9 buffer and desired volume of 1 mM vitamin B12 were added to these cultures to make 32, 64 and 128nM of vitamin B12-supplemented bacterial feed.

Propionate supplementation

Sodium propionate (P1880; Sigma, USA) stock concentration of 1 M (pH 7) was prepared in MilliQ water. The Sodium propionate stock was filtered using 0.2mm filter, further the filtered stock was added to NGM agar to prepare different concentrations (0, 110, 130, 150 mM) of propionate-supplemented media plates which were later seeded with overnight-grown bacterial lawns.

Choline supplementation

Choline chloride (A15828; Alfa Aesar, USA) stock concentration of 2 M was prepared in MilliQ water. The stock was filtered using 0.2mm filter, further the filtered stock was added to NGM agar to prepare different concentrations (0, 2, 5, 10, 20 and 40mM) of choline-supplemented media plates which were later seeded with overnight-grown bacterial lawns.

Life span analysis

Gravid worms of desired strain, grown on *E. coli* OP50, were bleached and eggs were subjected to L1 synchronization in 1 x M9 for 16 hours at 20 °C before growing the worms on respective RNAi, vitamin B12 supplemented and/or choline supplemented plates. Once the worms reached late L4 stage, they were transferred to respective plates overlaid with 5-fluorodeoxyuridine (FUDR, final concentration 0.1 mg/ml of media). Life span scoring was started on 7 th day of adulthood and continued every alternate day. For statistical analyses of survivability rate, OASIS software (http://sbi.postech.ac.kr/oasis) was used and *P*-values were calculated by applying a log rank (Mantel-Cox method) test. Unhealthy worms were censored.

Osmotic stress assay

Worms were grown till L4 stage on NGM plates (containing 50 mM NaCl) seeded with different bacterial feeds (supplemented or RNAi). L4 stage worms were the placed on unseeded NGM (n≈75 and N=3) plates containing 350 mM sodium chloride for 9 minutes. Post 9 mins, the worms were transferred onto unseeded NGM plates containing 50 mM NaCl. The fraction of motile worms was calculated over a time period of 15 minutes to determine percentage recovery. Statistical analyses were performed using Graphpad 9.0.

Propionate toxicity assay

L1 synchronized worms were placed on propionate-supplemented (0, 110, 130, 150 mM) plates seeded with *E. coli* OP50 or *E. coli* HT115. Worm development was observed till 72 h post L1 to check for developmental arrest at each stage. Un-arrested worms were counted, and imaging was performed using Axiocam MRm (Carl Zeiss, Germany) camera attached to M205FA microscope (Leica, Germany).

CyTP gene activation

For *Pcyp-35B1::GFP* expression studies, *bvIs5* and *flr-4*(*n2259*)X;*bvIs5* gravid worms (64-68 h post L1), subjected to different diet, RNAi or supplementation, were mounted on 2% agarose slides (n≈50, N=3) using 10 mM sodium azide. For *acdh-*

1p::GFP expression studies, *wwIs24* and *flr-4*(*n2259*)X;*wwIs24* worms (≈48 h post L1), grown on different bacterial feed, were mounted on 2% agarose slides (n≈50, N=3) using 10mM sodium azide. The GFP Fluorescence of worms were captured with the help of AxioImager M2 microscope (Carl Zeiss, Germany) fitted with Axiocam MRc at 10X magnification (Excitation 488nm and Absorbance at 520nm). The photos of individual samples of an experimental condition were stitched using Adobe Photoshop cs2 to get the entire panoramic representative image of individual experimental results. Quantification of GFP expression was done using NIH ImageJ software. Statistical analyses were done using Graphpad 9.0.

RNA isolation

Synchronized L1 worms grown on supplemented, or RNAi plates were collected on Day 1 of adulthood (64-68 h post L1) in 1 x M9 buffer and further washed three times in 1 x M9 buffer. Trizol of approximately 4 times the volume of the worm pellet was added, and the worms lysed using three freeze thaw cycles. Further, the worms were subjected to vigorous vortexing. RNA was isolated using phenol:chloroform:isoamylalcohol extraction followed by isopropanol precipitation. For quantitative Reverse Transcriptase PCR (qRT-PCR) experiments, the RNA concentrations were estimated using NanoDrop 2000 (Thermo Scientific, USA) and the integrity of the ribosomal 28 S and 18 S was determined using denaturing agarose gel.

QRT-PCR analysis

Approximately 1 µg of RNA was converted to cDNA with the help of Superscript III Reverse Transcriptase enzyme and poly-T primers (Invitrogen, USA). To determine the relative gene expression levels, QRT-PCR analysis was performed using the 2X Brilliant III Ultra-Fast SYBR® Green QPCR mastermix (Agilent Technologies, USA) and Agilent AriaMx Real-time PCR System (Agilent Technologies, USA). Statistical analysis was completed using Graphpad 9.0. Expression levels were normalized to actin.

Sr. No	Gene	Primer sequence
1	*acdh-1*	FP:- GCAAATGCAGATCCTAGCC<br>RP:- GTTTGTCTTCCTCCTTATCTACAG
2	*hphd-1*	FP:- GATATTCCGAACAATGCCAG<br>RP:- TCCAAAGTCTCGCATATAACC
3	*pmt-1*	FP:- CTTCTCTCTACATTAATCTTCTCCG<br>RP:- AGTGGACTTGACGCCAGTTGTT
4	*pmt-2*	FP:- GAGTTCATGTCGAAGTTTACCCAA<br>RP:- GGTTGTCCTTCTCGATGTATCC

Western blotting

Synchronized L1 worms (n≈50) were grown on respective bacterial feed, with or without supplementation. On Day 1 of adulthood (64 h-68 h), worms were collected in and washed thrice using 1 x M9 buffer. Further, the pellet was resuspended in 1X Laemmli Buffer (Tris-Cl, 0.2M pH 6.8; SDS, 10% w/v; glycerol 40% v/v; β-mercaptoethanol, 20% v/v; Bromophenol blue, 0.2% v/v) freeze-thawed 3 times and subjected to 95 °C for 10 mins. The samples were centrifuged at 13000 rpm at 4 °C for one minute. The supernatant was separated on a 10% SDS-PAGE and transferred to PVDF membrane. The membranes were subjected to blocking for 1 hour at room temperature in 5% BSA dissolved in 1X TBST (TBS with 0.1% Tween 20) and probed with primary anti-PMK-1 antibody (Cell Signaling Technology, USA; 1:10,000 dilution of 5% BSA in 1X TBST) or anti-phospho-PMK-1 antibody (Cell Signaling Technology, USA; 1:5000 dilution of 5% BSA in 1X TBST) or anti-actin antibody (Cell Signaling Technology, USA; 1:10000 dilution of 5% BSA in 1X TBST), incubated overnight (16 h) at 4°C. Next day, the membranes were washed thrice in 1X TBST for 10 mins each and incubated in secondary antibody of anti-rabbit conjugated to HRP (Cell Signaling Technology, USA; 1:10,000-dilution of 1X TBST) for 1 hr at room temperature. The blots were further washed 5-6 times with 1X TBST, for 5 mins each. The blots were developed with the help of chemiluminescent substrate (Millipore, USA).

For the quantification of p-PMK-1 and PMK-1 activity, the band intensities of total PMK-1 and pPMK-1 were quantified and compared with the basal intensity of beta-actin

bands using ImageJ software (National Institutes of Health, Bethesda, MD; http://rsb.info.nih.gov/ij/). The values obtained for pPMK-1 were divided by the values of total PMK-1 and provided as percentile representation. Statistical analysis was completed using Graphpad 9.0.

Measuring Intracellular Vitamin B12 concentrations of *C. elegans*

Intracellular vitamin B12 was measured by a kit-based electrochemiluminescence immunoassay (ECLIA) using COBAS e411 Analyzer. Briefly, *C. elegans* pellet from each of the conditions were taken in a microcentrifuge tube, washed three times with water and gently pelleted. A 10 mg freeze dried weight of pellet was then suspended in 300 µl of 1 x PBS, followed by homogenization using a hand-held homogenizer. The suspension was then centrifuged at 15,000 g at room temperature for 10 minutes. Supernatant was transferred to a fresh tube and subjected for vitamin B12 measurement. Vitamin B12 measurements were statistically analysed using GraphPad 9.0.

Measuring Intracellular Vitamin B12 concentrations of bacteria

Intracellular B12 was measured as above. Bacterial cells equivalent to OD_{600} 150 (OD_{600} = 1 ~ $3x10^{9}$ cells) were taken in a microcentrifuge tube, pelleted by centrifugation and washed three times with water. The pellet was then suspended in 500 µl of 1 x PBS, followed by lysis using a FastPrep-24™ 5G bead beater (MP Biomedicals, CA, USA). The suspension was then centrifuged at 15,000 g at 4 °C for 10 minutes. Supernatant was transferred to a fresh tube and subjected for vitamin B12 measurement. Vitamin B12 measurements were statistically analyzed was using Graphpad 9.0.

Metabolomics

Intracellular metabolites for MS-based targeted metabolomics were extracted using cold Actonitrile-methanol-water. Briefly, 10 mg dry weight of *C. elegans* pellet was collected by centrifugation in a microcentrifuge tube and washed three times with sterile water, then quenched with chilled Acetonitrile-methanol-water (3:5:2) (kept at −80°C), followed by sonication. The suspension was then transferred to a fresh tube and

centrifuged at 15,000 g at 4°C for 10 min. Supernatant was vacuum dried and reconstituted in 50 µl of 50% methanol. The reconstituted mixture was centrifuged at 15,000 g for 10 min, and 5 µl was injected for LC–MS/MS analysis.

The data were acquired using a Sciex Exion LCTM analytical UHPLC system coupled with a triple quadrupole hybrid ion trap mass spectrometer (QTrap 6500; Sciex) in a positive mode. Samples were loaded onto an Acquity UPLC BEH HILIC (1.7 µm, 2.1 × 100 mm) column, with a flow rate of 0.3 ml/min. The mobile phases comprising of 10 mM ammonium acetate and 0.1% formic acid (buffer A) and 95% acetonitrile with 5 mM ammonium acetate and 0.2% formic acid (buffer B). The linear mobile phase was applied from 95% to 20% of buffer A. The gradient program was used as follows: 95% buffer B for 1.5 min, 80%–50% buffer B in next 0.5 min, followed by 50% buffer B for next 2 min, and then decreased to 20% buffer B in next 50 s, 20% buffer B for next 2 min, and finally again 95% buffer B for next 4 min. Data were acquired using five biological replicates, with three technical replicates, for each run. Relative quantification was performed using MultiQuantTM software v.2.1 (Sciex).

Cysteine and Homocysteine were measured by HPLC (Agilent 1290 Infinity II LC system). Briefly 10mg dry weight of worms were suspended in 100µl of autoclaved milliQ water and lysed by 5 rounds of vortex-mixing with glass beads for 2 min each. The supernatant was then collected by centrifugation at 16000g for 10 minutes and the level of Cysteine and Homocysteine were determined using HPLC-FD. Briefly, 65 µl of this lysate was treated with 35µl of 1.43 M sodium borohydride in 0.10 M sodium hydroxide followed by the addition of 35µl of 1.0 M HCl. To this, 50µl of 7 mM mono bromobimane in 5 mM sodium EDTA (pH 7.0) was added and the solution was incubated at 42 °C for 12 mins followed by 45 minutes incubation in dark. Intracellular proteins were then precipitated by the addition of 50µl of 1.5 M $HClO_4$ followed by centrifugation at 16000g for 5 min. The supernatant was then transferred to vials for automated HPLC analysis. HPLC measurements were done by using reverse-phase C18 column (2.7µM bead size; 3.0 mm×100 mm, Agilent) with a flowrate of 0.7ml/min.

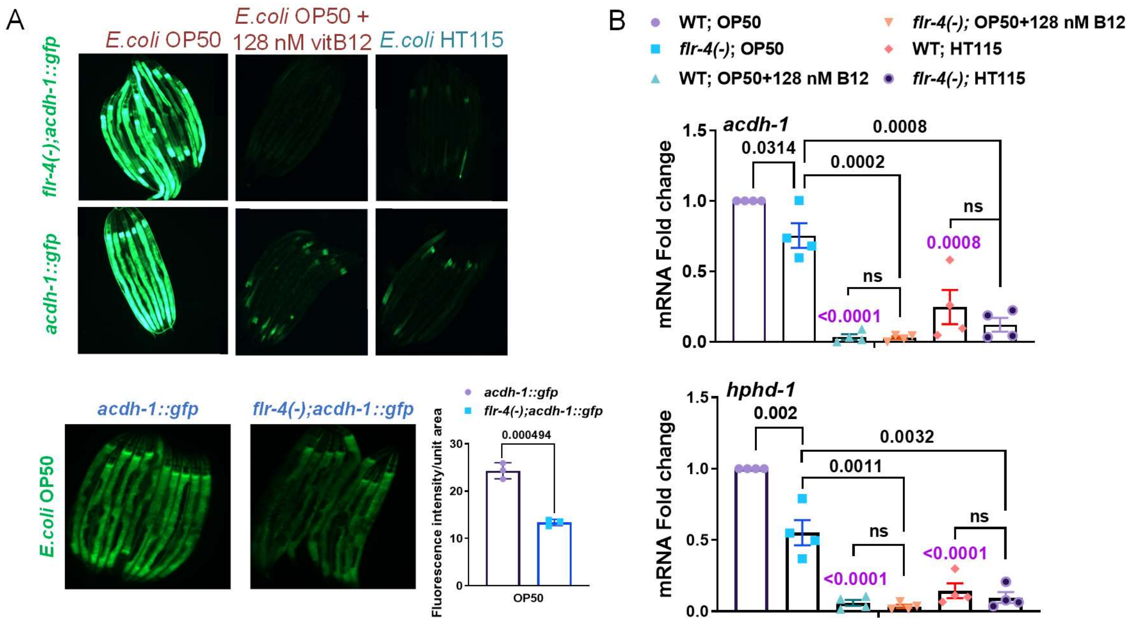

Supplementary Figure 1

A) Vitamin B12-responsive GFP expression in *acdh-1::gfp* and *flr-4(n2259);acdh-1::gfp* fed *E.coli* OP50 (2000 ms exposure time), OP50 supplemented with 128 nM Vitamin B12 and HT115 (2 s exposure time). The levels of *gfp* are higher on OP50, suggesting that this *E. coli* strain has lower Vitamin B12 levels. One of two biologically independent experiments shown. The images in the lower panel are taken with 90 ms exposure time so that the difference in fluorescence between *acdh-1::gfp* and *flr-4(n2259);acdh-1::gfp* is clearly visible. The quantification of the images from three biologically independent is shown. Data presented as mean value +/- SEM. Unpaired two-tailed *t*-test.

B) QRT-PCR showing that *acdh-1* and *hphd-1* genes of the non-canonical propionate breakdown pathway are down-regulated in wild-type and *flr-4(n2259)* grown on HT115 or OP50 supplemented with 128 nM Vitamin B12, compared to OP50. Expression levels were normalized to *actin*. Averages of four biological replicates are shown. Data presented as mean value +/- SEM. *ns* = non-significant. Unpaired two-tailed *t*-test. *P*-values in purple are in comparison to wild-type on OP50. The levels of these genes are significantly lower in *flr-4(n2259)* compared to wild-type, when both stains were grown on OP50.

Experiments were performed at 20 °C.

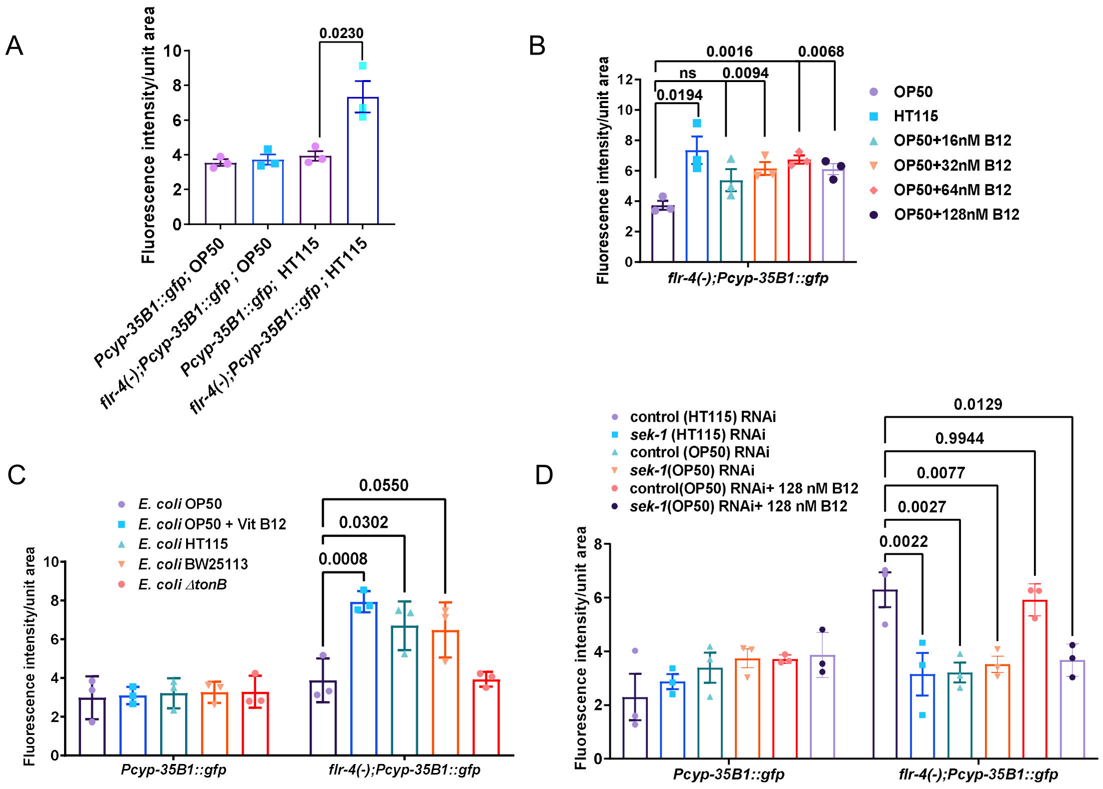

Supplementary Figure 2

A) Quantification of *gfp* expression in Figure 2A (upper panel). Average of three biological replicates (n≥50). Error bars are SEM. Unpaired two-tailed *t*-test. Each point represents fluorescence intensity/unit area of one biological replicate.

(B) Quantification of *gfp* expression in Figure 2A (lower panel). Average of three biological replicate (n≥50). Error bars are SEM. Unpaired two-tailed *t*-test. *ns* non-significant.

C) Quantification of *gfp* expression in Figure 2C. Average of three biological replicates (n≥50). Error bars are SEM. Two-way Annova. Each point represents mean fluorescence intensity/unit area of one biological replicate.

D) Quantification of *gfp* expression in Figure 2D. Average of three biological replicates (n≥50). Error bars are SEM. Two-way Annova. Each point represents mean fluorescence intensity/unit area of one biological replicate.

Experiments were performed at 20 °C. Source data is provided as a source data file.

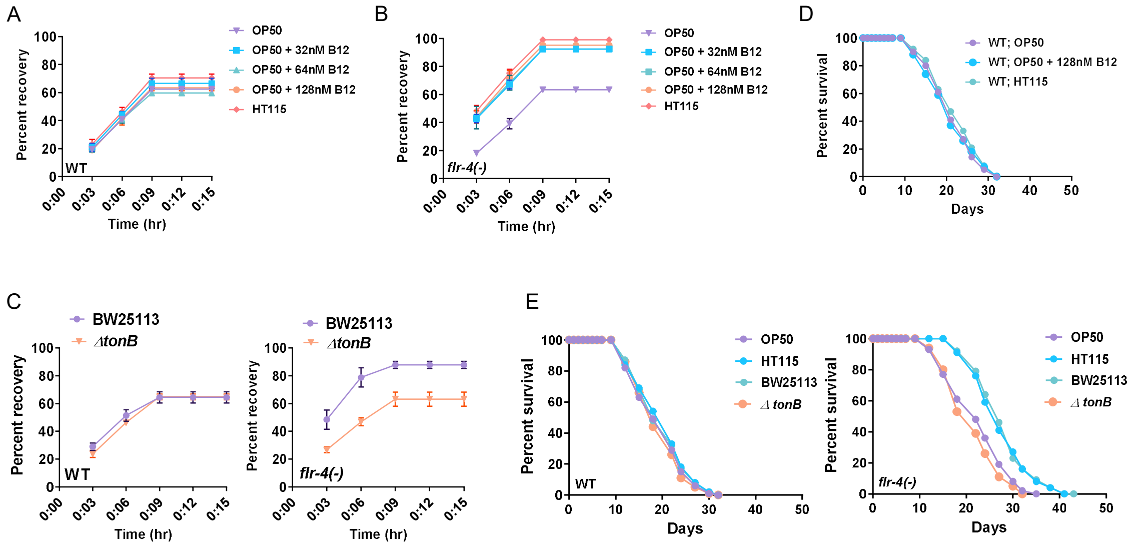

Supplementary Figure 3.

A) Vitamin B12 supplementation (32, 64 and 128nM) has little effect on osmotic tolerance of wild-type worms.

B) Vitamin B12 supplementation (32, 64 and 128nM) increased osmotic tolerance of *flr-4(n2259)* worms.

C) The wild-type worms do not exhibit difference in osmotic stress tolerance on *E. coli* BW25113 or *ΔtonB* (Left panel). The *flr-4(n2259)* worms exhibit more osmotic stress tolerance on *E. coli* BW25113 compared to *ΔtonB* (Right panel).

D) No difference in life span was observed when wild-type was grown on OP50, HT115 or OP50 supplemented with 128nM Vitamin B12.

E) The *flr-4(n2259)* worms live long on *E. coli* BW25113 but not on *ΔtonB* (Right panel). No difference is observed in wild-type worms (Left panel).

Life span and osmotic tolerance assays were performed at 20 °C. One of three biologically independent experiment shown. Life span summary is provided in Supplementary Table 1. Summary of osmotic tolerance assay is provided in Supplementary Table 2.

Source data is provided as a source data file.

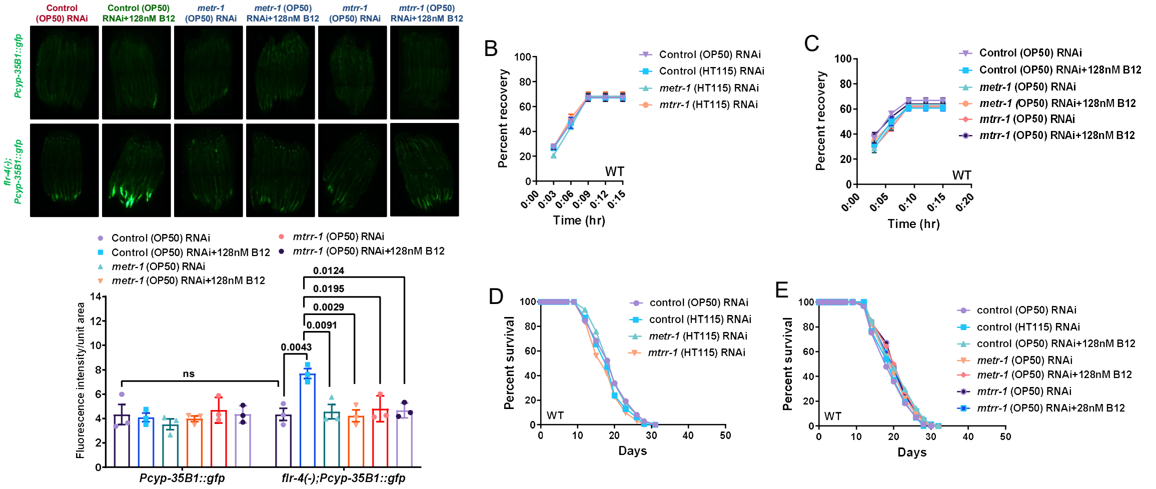

Supplementary Figure 4.

A) (Upper panel) The increased expression of *gfp* that is observed in *flr-4*(*n2259);Pcyp-35B1::gfp* grown on control (OP50) RNAi supplemented with Vitamin B12 is suppressed when the strain is grown on *metr-1* or *mtrr-1* (OP50) RNAi background. One of three biological replicates. (Lower panel) Quantification of upper panel. Average of 3 biologically independent experiments ± SEM. *P*-value determined using two-way Annova. Ns = non-significant.

B) Wild-type worms have similar recovery kinetics when *metr-1* or *mtrr-1* was knocked down using (HT115) RNAi. One of three biological replicates shown.

C) Wild-type worms have similar recovery kinetics when *metr-1* or *mtrr-1* was knocked down using (OP50) RNAi in presence or absence of 128 nM Vitamin B12. One of three biological replicates shown.

D) Wild-type worms have similar life spans when *metr-1* or *mtrr-1* was knocked down using (HT115) RNAi. One of three biological replicates shown.

E) Wild-type worms have similar life spans when *metr-1* or *mtrr-1* was knocked down using (OP50) RNAi in presence or absence of 128 nM Vitamin B12. One of three biological replicates shown.

All experiments were performed at 20 °C. Life span summary is provided in Supplementary Table 1. Summary of osmotic tolerance assay is provided in Supplementary Table 2.

Source data is provided as a source data file.

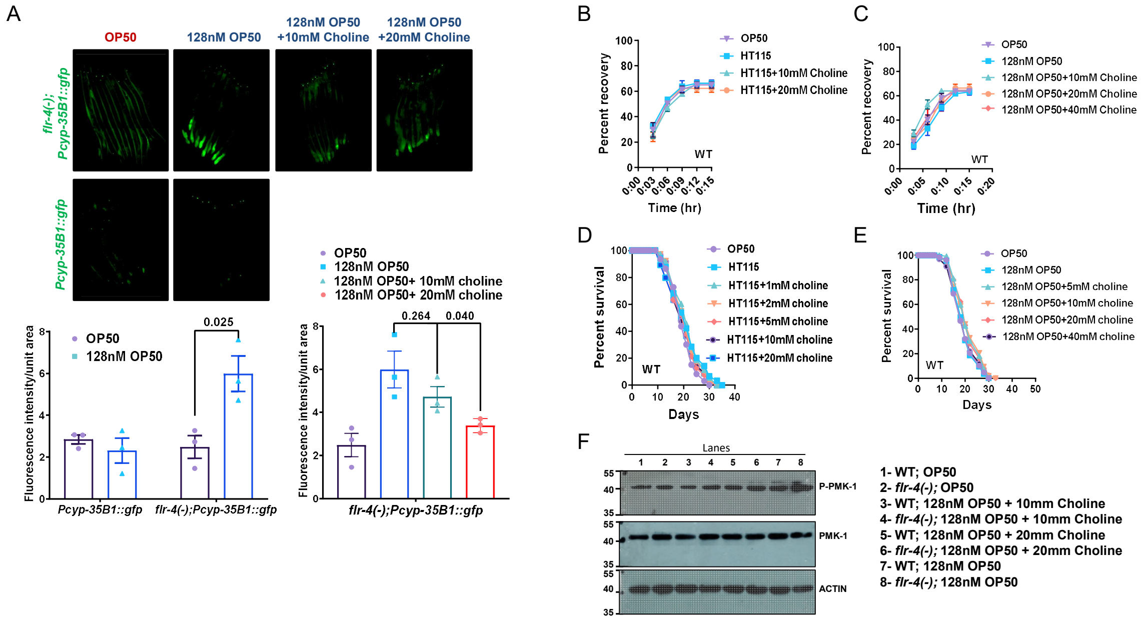

Supplementary Figure 5

A) The increased expression of *gfp* in *flr-4*(*n2259);Pcyp-35B1::gfp* grown on OP50 supplemented with 128 nM Vitamin B12 (as compared to OP50) is suppressed when the strain is grown on different concentrations of choline (10 or 20 mM). Quantification of images is shown below. Error bars are SEM. *P*-values determined by Unpaired *t*-test.

B) The wild-type worms do not exhibit osmotic stress tolerance when grown on HT115, as compared to OP50. The supplementation of choline (10 or 20 mM) does not affect osmotic stress tolerance in wild-type worms.

C) The wild-type worms do not exhibit osmotic stress tolerance when grown on OP50 supplemented with 128 nM Vitamin B12, as compared to OP50. The supplementation of choline (10, 20 or 40 mM) does not affect osmotic stress tolerance in wild-type worms.

D) The wild-type worms do not exhibit increased life span when grown on HT115, as compared to OP50. The supplementation of choline (1, 2, 5, 10 or 20 mM) to HT115 does not affect life span in wild-type worms.

E) The wild-type worms do not exhibit increased life span when grown on OP50 supplemented with 128 nM Vitamin B12, as compared to OP50. The supplementation of choline (5, 10, 20 or 40 mM) does not affect life span in wild-type worms.

F) Representative western blot of wild-type and *flr-4(n2259)* worms grown on OP50 or OP50+128 nM Vitamin B12, with or without choline supplementation, and probed with anti-

phospho-PMK-1 (P-PMK-1), anti-PMK-1 or anti-Actin antibodies. One of three biologically independent experiments shown.

One of three biologically independent replicates shown for all experiments. Life span and osmotic stress tolerance assays were performed at 20 °C. Life span summary is provided in Supplementary Table 1. Summary of osmotic tolerance assay is provided in Supplementary Table 2.

Source data is provided as a source data file.

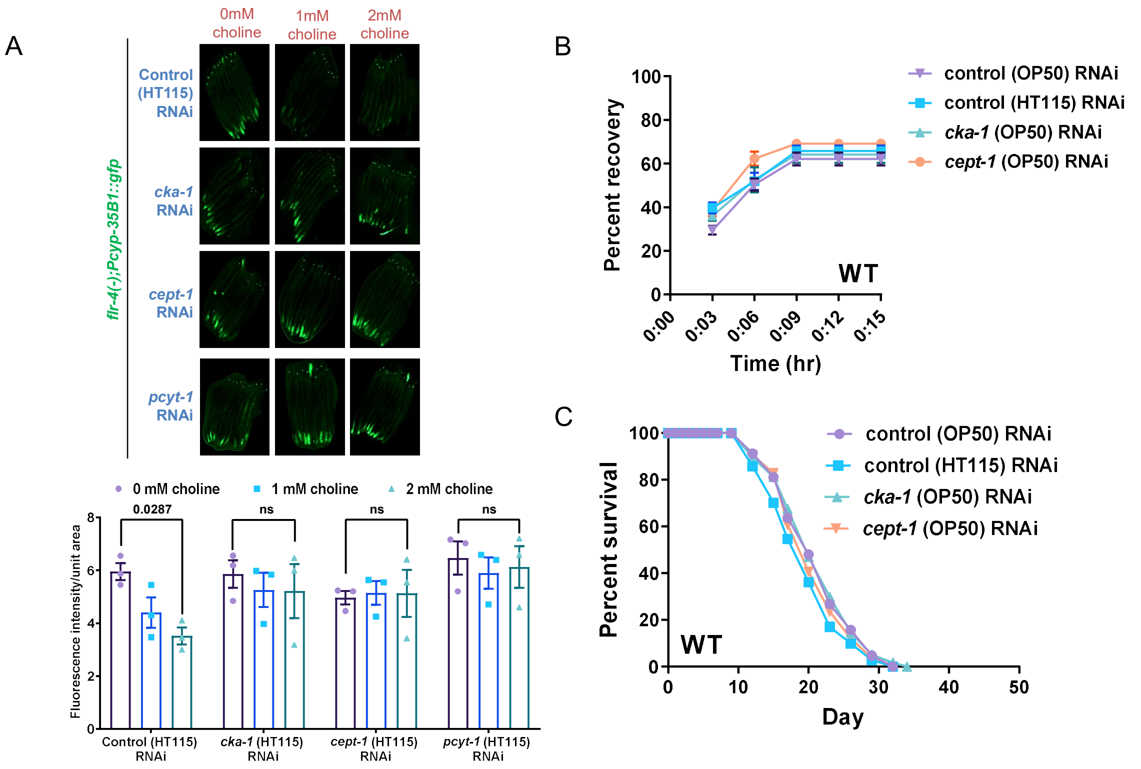

Supplementary Figure 6

A) The expression of *gfp* in *flr-4(n2259);Pcyp-35B1::gfp*, when grown on control (HT115) RNAi, is suppressed upon choline supplementation (1 and 2 mM). However, when *cka-1*, *pcyt-1* or *cept-1* is knocked down using (HT115) RNAi, choline is not able to suppress *gfp* expression. Quantification of three biologically independent replicates is provided in the bottom panel. Two-way Annova. Error bars are SEM.

B) No difference in osmotic stress tolerance is observed in wild-type grown on control (HT115) RNAi, control (OP50) RNAi or on knocking down *cka-1* and *cept-1* using an OP50 RNAi system.

C) No difference in life span is observed in wild-type grown on control (HT115) RNAi, control (OP50) RNAi or on knocking down *cka-1* and *cept-1* using an OP50 RNAi system.

One of three biologically independent replicates shown for all experiments. All experiments were performed at 20 °C. Life span summary is provided in Supplementary Table 1.

Summary of osmotic tolerance assay is provided in Supplementary Table 2.

Source data is provided as a source data file.

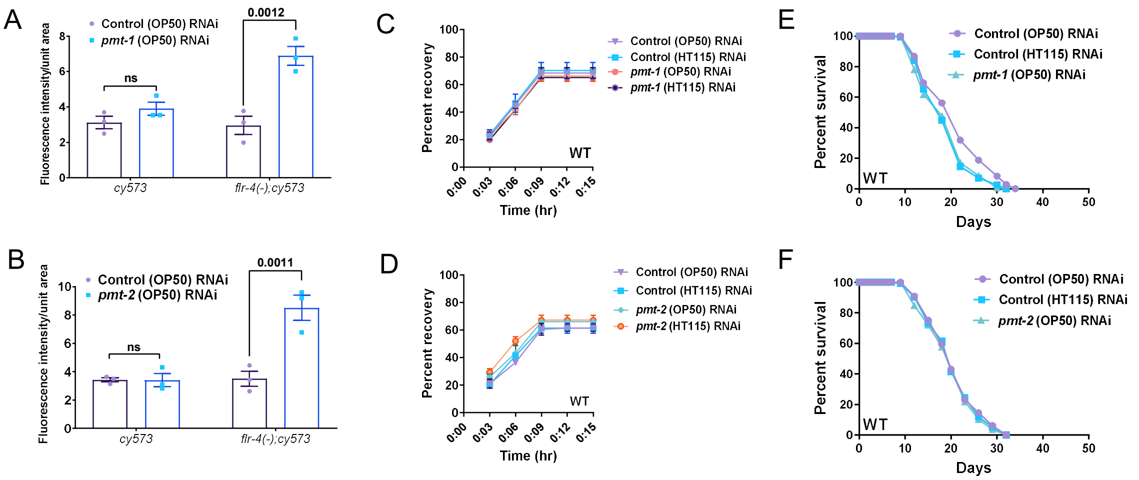

Supplementary Figure 7.

A) Quantification of fluorescence on *pmt-1* RNAi in Figure 7B. Mean of three biologically independent experiments ± SEM. Two-way Annova. ns = non-significant

B) Quantification of fluorescence on *pmt-2* RNAi in Figure 7B. Mean of three biologically independent experiments ± SEM. Two-way Annova. ns = non-significant

C-D) The osmotic tolerance of wild-type worms are unaffected on *pmt-1* and *pmt-2* RNAi. One of three biologically independent replicates shown.

E-F) The life span of wild-type worms is unaffected on *pmt-1* and *pmt-2* RNAi. One of three biologically independent replicates shown for all experiments.

Experiments were performed at 20 °C. Summary of osmotic tolerance assay is provided in Supplementary Table 2. Source data is provided as a source data file.
